# Sindbis virus interferes with dengue 4 virus replication and its potential transmission by A*edes albopictus*

**DOI:** 10.1186/s13071-015-0667-y

**Published:** 2015-01-30

**Authors:** Ephantus J Muturi, Jeffrey Bara

**Affiliations:** Illinois Natural History Survey, Prairie Research Institute, University of Illinois at Urbana-Champaign, Champaign, Illinois 61820 USA

**Keywords:** Dengue virus, Sindbis virus, Coinfection, Superinfection, Cell culture, *Aedes albopictus*

## Abstract

**Background:**

Mosquitoes transmit a number of arboviruses associated with disease outbreaks in humans and other animals. The majority of medically important arboviruses belong to three families: *Togaviridae*, *Flaviviridae* and *Bunyaviridae*. Several members of these families have overlapping distributions and share common vectors, increasing the potential for arboviral coinfections. This study examined how two model viruses: Sindbis virus (SINV, *Togaviridae: Alphavirus*) and dengue-4 virus (DENV-4, *Flaviviridae: Flavivirus*) may interact in C6/36 *Aedes albopictus* cells and in the mosquito vector *Ae. albopictus*.

**Methods:**

C6/36 cells were coinfected, superinfected, or singly infected with SINV and DENV-4 and the two viruses quantified at different time points. Four to seven day old adult females of *Ae. albopictus* were also fed blood containing one or both viruses and viral infection and dissemination rates determined.

**Results:**

Sindbis virus suppressed replication of DENV-4 in C6/36 *Ae. albopictus* cells with greater inhibition occurring when the two arboviruses were inoculated simultaneously compared to sequentially. In addition, *Ae. albopictus* simultaneously exposed to both arboviruses had significantly lower DENV-4 infection and population dissemination rates compared to those exposed to DENV-4 alone.

**Conclusion:**

These results suggest that certain *Alphaviruses* may interfere with DENV-4 transmission by suppressing its replication and increasing vector refractoriness. The findings provide important insights into the potential contribution of mixed arboviral infections to DENV transmission dynamics.

## Background

Mosquito-borne viral diseases are a serious threat to human, veterinary, and wildlife health, and constitute a significant fraction of the global infectious disease burden. In the last few decades, arboviruses have emerged in places with no previous history of activity and re-emerged in regions where they had previously been controlled or eradicated, primarily due to globalization and climate change [1]. The majority of medically important mosquito-borne viruses belong to three families: *Flaviviridae* (e.g. dengue (DENV), yellow fever (YFV)), *Togaviridae* (e.g. Chikungunya (CHIKV), Eastern equine encephalitis (EEEV)) and *Bunyaviridae* (e.g. the California group viruses such as La Crosse virus (LACV)). Several members of these families have overlapping distributions and complete their life cycle in the same vertebrate and mosquito hosts. As a result, mixed infections of mosquito-borne viruses are fairly common [2-5].

Mixed infections can be broadly classified into two categories: coinfection and superinfection [6]. In coinfection, two or more viruses invade the host/vector simultaneously or in a short time interval whereas in superinfection, different viruses (strains) invade the host at different times. Mixed arboviral infections may generally interact synergistically where at least one virus facilitates replication or transmission of the other virus, or antagonistically where one virus benefits and its presence and activity reduces the fitness of the second virus. These interactions have been documented in mosquito-borne viruses both *in vitro* [7-11] and *in vivo* [12-14]. Viral interference (inhibition of virus growth by another) appears to be the most common form of interaction and is more pronounced when the interfering virus is introduced prior to or at a higher multiplicity of infection relative to the challenge virus [7,10,11]. However, viral facilitation is also possible [14,15]. Viral interference may occur either extracellularly during the early stages of attachment and penetration where virus-induced alterations in cell membranes prevent the second virus from interacting normally with the cell surface, or intracellularly where the interfering virus successfully competes for limited components essential for virus replication [7,8,16]. Genetic reassortment resulting in variants showing novel genetic features is also a well-documented outcome of arbovirus interactions and is often responsible for major genetic shifts in viral populations within hosts/vectors [17,18]. Accurate knowledge on how mixed viral infections interact within the vertebrate hosts and insect vectors is therefore essential for understanding of viral pathogenesis and evolution and for the development of efficient and stable control strategies. However, little is known regarding how different mosquito-borne viruses interact within their shared vectors.

The objective of this project was to determine whether infection with an *Alphavirus* alters vector susceptibility to DENV. We addressed this objective by infecting *Aedes albopictus* mosquito (C6/36) cells and *Ae. albopictus* mosquitoes with Sindbis virus (SINV, *Togaviridae*: *Alphavirus*) and DENV-4 in single and dual infection treatments. The cells were infected with the two viruses either individually (single infection), simultaneously (coinfection) or at different times (superinfection) and virus titers quantified every 12 hours for 5.5 days. *Ae. albopictus* were orally challenged with DENV-4 and SINV in single and coinfection treatments and effects on DENV-4 and SINV infection and dissemination rates determined. DENV-4 is one of the four serotypes of DENV, the causative agents of dengue and dengue hemorrhagic fever. Over the last few decades the global incidence of DENV has increased dramatically and is currently considered as the most important arboviral disease of human [19]. Coinfections of each of the four DENV serotypes with CHIKV have been reported in humans [3,4]. Thus the choice of DENV-4 in this study was based solely on its availability at the time of initiating the experiments. SINV is an excellent model for understanding how medically important *Alphaviruses* such as CHIKV, which has caused severe epidemics in Africa, Europe and Asia [20], may interact with DENV and other *Flaviviruses* within the vector. This virus is readily transmitted by *Aedes* mosquitoes under laboratory conditions [21], and unlike CHIKV, it can be manipulated under biosafety level 2 arthropod containment facility. We tested the hypotheses that: 1) SINV alters replication of DENV-4 in C6/36 cells, and 2) *Ae. albopictus* simultaneously exposed to SINV and DENV-4 are more or less competent for DENV-4 relative to those exposed to DENV-4 alone.

## Methods

### Cells and viruses

The C6/36 *Aedes albopictus* epithelial cells (ATCC CRL-1660) were maintained at 32°C in Leibovitz L-15 media (Invitrogen, Carlsbad, CA), supplemented with 10% fetal bovine serum (FBS; Atlanta Biological, Norcross GA) and 1% penicillin/streptomycin (Invitrogen). The model viruses were SINV strain MRE16 from Sindbis Health District, Nile Delta, Egypt and DENV-4 strain P84.

### Virus growth in cell cultures

To test how sequential (superinfection) and simultaneous (coinfection) infections of SINV and DENV-4 affect virus replication, confluent monolayers of C6/36 cells in 25 cm^2^ flasks were rinsed with 3 ml of phosphate buffered saline (PBS) and inoculated with one or both viruses at multiplicity of infection (MOI) of either 0.1 or 0.006 (Table 1). Two types of mixed infections were established: coinfections, where both viruses were inoculated in C6/36 cells simultaneously and superinfections, where the second virus was added 2 hours after the first virus (Table 1). Briefly, C6/36 monolayers were inoculated with 250 μL of media containing SINV, DENV-4, or both viruses and incubated at 32°C for 1 hour with intermittent rocking of the flasks to enable adsorption of the virus. The flasks were then replenished with 5 ml of fresh L-15 media and incubated for 1 hour. After this period, the media was removed from flasks and the monolayers rinsed with 3 ml of PBS before inoculating them with 250 μL of SINV or DENV-4 for superinfection treatments, or a sham treatment containing 250 μL of L-15 media (without the virus) for single infection and coinfection treatments using the procedures described above. The flasks were incubated at 32°C and viral replication monitored every 12 hours for 5.5 days. To accomplish this, 300 μl of media was collected with replacement from each flask and preserved at −80°C for later quantification of the two viruses using real-time quantitative reverse transcription polymerase chain reaction (qRT-PCR) as described below. At 72 hours post inoculation, images of each cell monolayer were photographed at 20X magnification using Cellsens digital imaging software (Olympus, Center Valley, PA). Each treatment had 3 biological replicates.

**Table 1:** **Experimental design for SINV and DENV-4 single infections, coinfections and superinfections in C6/36 mosquito cell lines**

	**Infection order**		**Infection MOI**	
**Infection type**	**First (0 hour)**	**Second (2 hour)**	**0 hour**	**2 hour**
Single	SINV	-	0.1	-
	SINV	-	0.006	-
	DENV-4	-	0.1	-
	DENV-4	-	0.006	-
Superinfection	SINV	DENV-4	0.1	0.1
	DENV-4	SINV	0.1	0.1
Coinfection	DENV-4 + SINV	-	0.1 + 0.1 (0.2)	-
	DENV-4 + SINV	-	0.1 + 0.006 (0.1006)	-
	DENV-4 + SINV	-	0.006 + 0.1 (0.1006)	-

Dash (-) indicates that uninfected media was added instead of virus-infected media.

To quantify virus growth in the cells, 220 μL of harvested media was used for total RNA extraction using Qiamp virus Biorobot 9604 kit according to manufacturer’s protocol (Qiagen, Valencia, CA). Total RNA was quantified using a nanodrop and 100 μL aliquots containing 2000 ng of total RNA were used as the source of RNA for SINV and DENV quantification by Taqman probe qRT-PCR. PCR amplification was conducted in 20 μL reactions containing 10 μL of one-step Sensifast RT-PCR master mix (Bioline, Tauton, MA ), 0.2 μL RNAse inhibitor, 0.8 μL of each 10 μM forward and reverse primer stock, 0.4 μL of 10 μM Taqman probe, 2.8 μL nuclease-free water (Integrated DNA Technologies, Coralville IA) and 5 μL template RNA (20 ng/μL). SINV primers and probe targeted the nonstructural protein 1; forward primer (5′-CACWCCAAATGACCATGC-3′), reverse primer (5′ -KGTGCTCGGAAWACATTC-3′), and probe (5′ FAM-CAGAGCATTTTCGCAT CTGGC-BHQ1-3′). DENV-4 primers and probe targeted the pre-membrane (prM) gene; forward primer (5′-TTGTCCTAATGATGCTGGTCG-3′), reverse primer (5′-TCCACCTGAGACTCCTTCCA-3′), and probe (5′ -JOE-TTCCTACTCCTACGCATCGCATTCCG-BHQ1-3′). qRT-PCR reactions were conducted in a 7300 real time PCR system (Applied Biosystems, Foster City, CA). Thermocycling conditions for SINV were: 50°C for 30 min, 95°C for 5 min followed by 35 cycles of 95°C for 30 s, 50°C for 30 s, and 72°C for 1 min. Thermocycling conditions for DENV-4 were: 50°C for 30 min, 95°C for 1 min followed by 45 cycles of 95°C for 15 s, and 60°C for 30 s. All qRT-PCR reactions were performed in triplicate.

Data analysis was conducted using SPSS version 21 (IBM, Armonk, NY) statistical package. Data were checked for normality and homogeneity of variances before conducting statistical analysis. SINV and DENV-4 titers were log transformed to improve normality before conducting statistical analyses. For each virus, one-way repeated measures ANOVA (RMANOVA) was used to determine the effect of inoculation treatment and incubation time on viral titer. When significant treatment effects were detected, pairwise differences between treatment means were determined using a Bonferroni adjustment for multiple comparisons.

### Vector competence studies

The experiment was conducted using F_18_ generation of *Ae. albopictus* originally from Vero Beach, Florida. First-instar larvae (<24 hr old) were added to 1.6 liters of filtered oak infusion held in 5-liter plastic containers at initial larval densities of 150 per container. The containers were maintained at 28°C and a 12:12 light:dark regime. Each container was supplemented with 0.2 and 0.05 g of larval food (1:1 albumin:yeast) on days 1 and 7, respectively. Pupae from each replicate were removed daily and placed into plastic vials with water until eclosion. Eclosing adults (both males and females) from each container were housed in paperboard cages (11 cm high × 9.5 cm diameter) according to the date of emergence and provided continuous access to 20% sucrose solution. There were 4 replicates for each treatment.

Four to seven day old females that had been sugar-starved for 48 hours were provided 40 minutes access to infectious blood meals containing single or coinfections of DENV-4 and SINV via the Hemotek membrane feeding system (Lancashire, UK). SINV infectious blood meal was prepared by adding 250 μL of a previously frozen stock virus to a 1:1 mixture of freshly harvested uninfected media and citrated bovine blood for a final titer of 10^5.1^-10^5.25^ plaque forming units per mL (pfu/mL). These titers were determined by plaque assays of 10-fold serial dilutions of SINV inoculated in confluent monolayers of African green monkey kidney (Vero) cells (ATCC CCL-81). Unlike DENV, SINV thawed from frozen stock is relatively efficient at infecting the mosquitoes [21,22]. DENV-4 infectious blood meal was prepared by adding 250 μL of uninfected media to a 1:1 mixture of freshly harvested DENV-4 and citrated bovine blood for a final titer of 10^7.4^ -10^7.9^ focus forming units per mL (ffu/mL). This virus (DENV-4) was obtained by inoculating confluent monolayers of C6/36 cells at a MOI of 0.1 and amplifying the virus for 5 days. The final working stock for DENV-4 was obtained by pooling freshly harvested virus-infected media from multiple flasks. The infectious blood meal for coinfection treatments was prepared by adding 250 μL of a previously frozen SINV stock virus to the 1:1 mixture of DENV-4 (described above) and citrated bovine blood in order to achieve the same titers used in single infection studies. For all treatments, equal volumes of media and blood were used to prepare the infectious blood meals. Blood meal titers for DENV-4 were determined by incubating confluent monolayers of Vero cells inoculated with 10-fold serial dilutions of DENV-4 for 5 days at 37°C and visualizing the foci by immunostaining [23]. Blood meal titers for DENV-4 coinfection treatment could not be determined due to cytopathic effects of SINV on Vero cells. However, since a single working stock was used for both coinfection and single infection treatments, it is reasonable to assume that the blood meal titers for these treatments were identical.

Blood-fed females were transferred into paperboard cages and maintained on 10% sucrose solution at 28°C and 70% relative humidity for a predetermined incubation period of 6, 9, or 12 days. After this incubation period, individual females were killed by freezing and dissected to remove their legs for disseminated infection assays. The bodies and legs were preserved separately at −80°C and later assayed for DENV-4 and SINV.

Bodies and legs of individual mosquitoes were homogenized in 1 ml of L-15 media and total RNA was extracted as described above. The samples were then assayed for DENV-4, SINV or both viruses using the primers, probes, and experimental procedures described above. Females with virus-positive bodies and legs were considered to have a disseminated infection, while those with an infected body and uninfected legs represented a non-disseminated infection. Infection rate was computed by dividing the number of mosquitoes with virus-positive bodies by the total number of mosquitoes that obtained an infectious bloodmeal. Dissemination rate was calculated as the proportion of mosquitoes with virus-positive legs out of the total number with virus-positive bodies. Population dissemination rate was calculated as the proportion of mosquitoes with virus-positive legs out of the total number that took an infectious bloodmeal.

Data analysis was conducted using SAS version 9.3 (SAS Inc., Cary, NC) statistical package. Due to uneven blood feeding success among replicates, adult females from all replicates of a treatment were pooled and chi square test was used to determine the effect of viral coinfection on midgut infection rates, dissemination rates and population dissemination rates 6, 9 and 12 days post exposure.

## Results

### Virus replication in cell cultures

**Figure 1:**
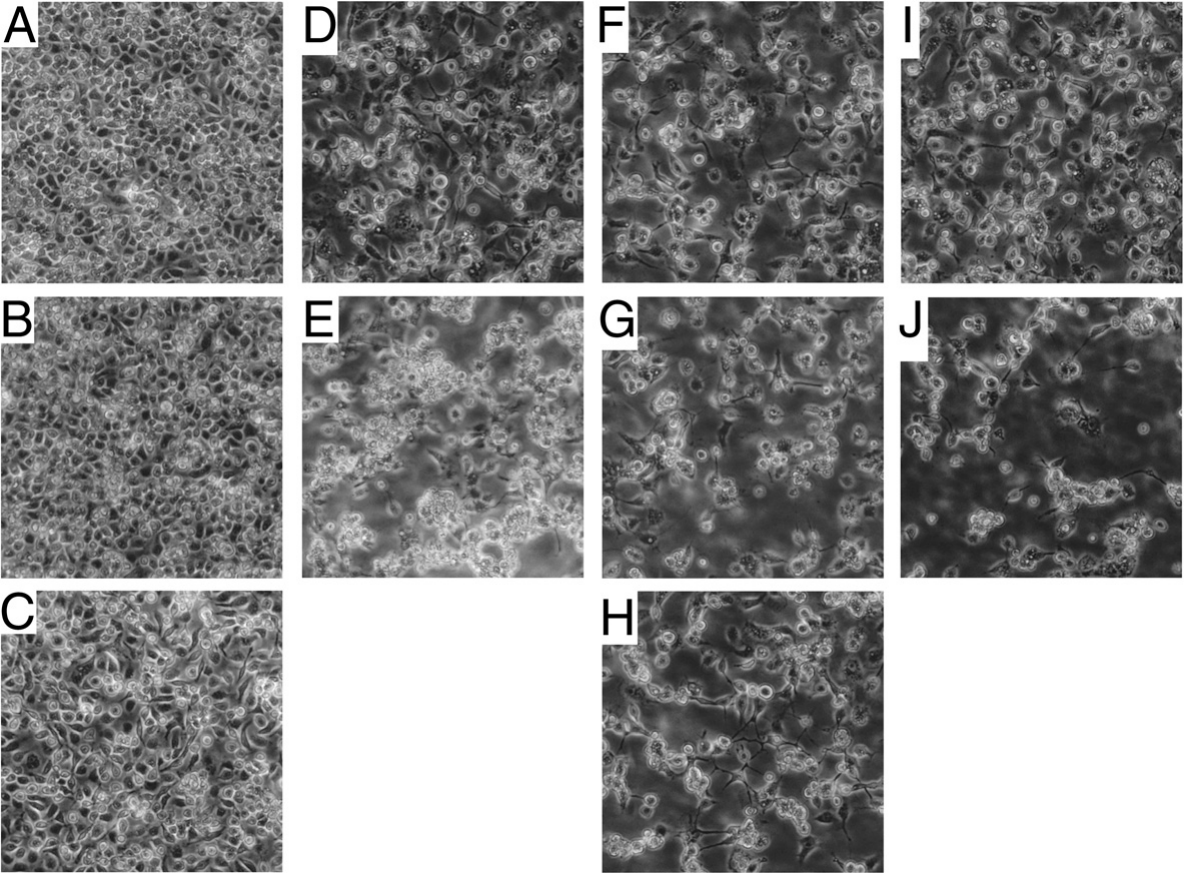
**Characteristics of C6/36 cells 72 hours after inoculation with DENV-4, SINV or both viruses. A)** control, **B)** DENV-4 alone at multiplicity of infection (MOI) of 0.1, **C)** DENV-4 alone at MOI of 0.006, **D)** SINV alone at MOI of 0.1, **E)** SINV alone at MOI of 0.006, **F)** DENV-4-SINV coinfection with each virus inoculated at MOI of 0.1 (1:1), **G)** DENV-4-SINV coinfection with DENV-4 inoculated at MOI of 0.006 and SINV inoculated at MOI of 0.1 (1:15), **H)** DENV-4-SINV coinfection with DENV-4 inoculated at MOI of 0.1 and SINV inoculated at MOI of 0.006 (15:1), **I)** DENV-4 was inoculated 2 hours ahead of SINV and each virus was inoculated at MOI of 0.1, and **J)** SINV was inoculated 2 hours ahead of DENV-4 and each virus was inoculated at MOI of 0.1.

There was no evidence of cytopathic effects in C6/36 cells inoculated with DENV-4 alone (Figure 1). However, cytopathic effects were observed in C6/36 cells inoculated with either SINV alone or SINV and DENV-4 coinfections and superinfections at both low and high virus titer (Figure 1).

**Figure 2:**
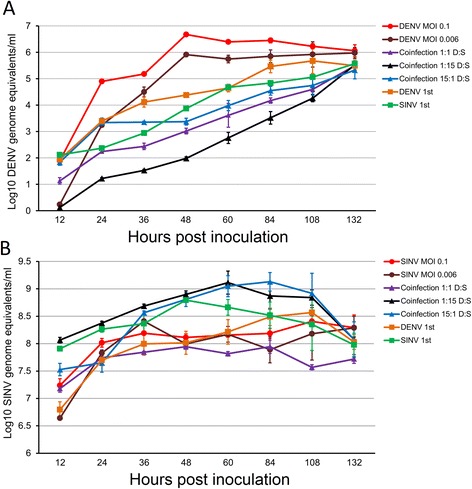
**Growth characteristics of A) DENV-4 and B) SINV among single infection, coinfection, and superinfection treatments.** The viruses were inoculated at MOI of 0.1 or 0.006. Error bars represent the standard errors.

**Figure 3:**
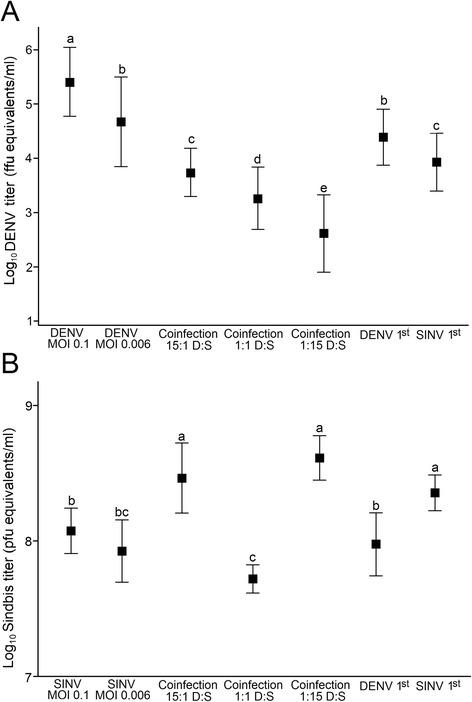
**Average titers for A) DENV-4 and B) SINV among single infection, coinfection, and superinfection treatments.** Values in different letters are significantly different at *P* < 0.05. Error bars represent the standard errors.

Coinfection and superinfection had significant effects on both DENV-4 and SINV replication (Table 2). DENV-4 virus titers increased over time across all treatments with the highest titers occurring among single infection treatments compared to dual infection treatments (i.e. coinfection and superinfection treatments) and among superinfection treatments compared to coinfection treatments (Figure 2). Among coinfection treatments, DENV-4 titers were lowest in 1:15 DENV-4: SINV coinfection treatment followed by 1:1 DENV-4:SINV and 15:1 DENV-4:SINV coinfection. Among superinfection treatments, DENV-4 titers were higher when it was inoculated before compared to after SINV inoculation (Figure 2). The average titers for DENV-4 over the 132 hour-period varied significantly among the seven treatments (Figure 3). The highest DENV-4 titers were observed when DENV-4 was inoculated singly at high MOI (0.1) followed by low MOI (0.006) single infection treatment and DENV-4 first superinfection treatment. Among the remaining treatments, DENV-4 titers were highest among 15:1 DENV-4:SINV coinfection treatment and SINV first superinfection treatment, intermediate in 1:1 DENV-4:SINV coinfection treatment and lowest in 1:15 DENV-4:SINV treatment.

**Table 2:** **RMANOVA for the effect of coinfection and superinfection on replication of dengue-4 and Sindbis viruses in C6/36 cells after correcting for multiple comparisons**

**Virus**	**Time (hours)**	**F value**	**df**	***P***
Dengue 4	12	202.64	6, 14	<0.001
	24	385.61	6, 14	<0.001
	36	405.36	6, 14	<0.001
	48	266.24	6, 14	<0.001
	60	68.71	6, 14	<0.001
	84	90.08	6, 14	<0.001
	108	26.77	6, 14	<0.001
	132	4.64	6, 14	0.01
Sindbis	12	41.22	6, 14	<0.001
	24	30.17	6,14	<0.001
	36	12.44	6, 14	<0.001
	48	37.96	6,14	<0.001
	60	19.71	6, 14	<0.001
	84	19.96	6,14	< 0.001
	108	22.58	6, 14	< 0.001
	132	8.36	6,14	0.001

In contrast, SINV titers at different post infection time points exhibited a pattern different from that of DENV-4 (Figure 2). Virus titers increased over the first 36 hours post infection before exhibiting a treatment-specific pattern thereafter (Figure 2). The 1:15 and 15:1 DENV-4:SINV coinfection treatments had the highest SINV titers between 36 and 108 hours post infection while the 1:1 DENV-4:SINV coinfection had the lowest SINV titers across time points (Figure 2). The average SINV titers were highest when SINV was inoculated ahead of DENV-4 or simultaneously with DENV-4 at 1:15 or 15:1 (DENV:SINV), intermediate when SINV was either inoculated singly (irrespective of MOI) or after DENV-4 inoculation, and lowest in the 1:1 DENV-4:SINV coinfection treatment (Figure 3).

### DENV-4 infection and dissemination rates in *Aedes albopictus*

At all 3 post infection incubation times (6, 9 and 12 days), DENV-4 infection rates were significantly higher among mosquitoes exposed to DENV-4 alone compared to those exposed to DENV-4 and SINV (6 dpe: χ^2^ = 5.5, df = 1, *P* = 0.019, 9 dpe: χ^2^ = 3.9, df = 1, *P* = 0.05, 12 dpe: χ^2^ = 13.4, df = 1, *P* < 0.001, Table 3). Among mosquitoes exposed to DENV-4 alone, days post exposure (dpe) had no significant effect on DENV-4 infection rates (χ^2^ = 0.74, df = 2, *P* = 0.69, Table 3). However, DENV-4 infection rates among mosquitoes coinfected with DENV-4 and SINV were 2 fold higher at 6 and 9 dpe compared to 12 dpe (Table 3).

**Table 3:** **Midgut infection rates, dissemination rates, and population dissemination rates for dengue-4 and Sindbis viruses among** ***Aedes albopictus*** **females exposed to the two viruses either individually or simultaneously**

		**Infection rates**			**Dissemination rates**			**Population dissemination rates**		
**Virus**	**Treatment**	**6 dpe**	**9 dpe**	**12 dpe**	**6 dpe**	**9 dpe**	**12 dpe**	**6 dpe**	**9 dpe**	**12 dpe**
DENV-4	Dengue alone	0.42 (91)	0.36 (67)	0.37 (90)	0.11 (38)	0.46 (24)	0.45 (33)	0.04 (91)	0.16 (67)	0.17 (90)
	Coinfection	0.25 (91)	0.22 (86)	0.12 (76)	0.22 (23)	0.16 (19)	0.44 (9)	0.05 (91)	0.03 (86)	0.05 (76)
SINV	SINV alone	0.04 (74)	0.08 (79)	0.0 (77)	0.0 (3)	0.0 (6)	0.0 (0)	0.0 (74)	0.0 (79)	0.0 (77)
	Coinfection	0.0 (91)	0.01(86)	0.04(76)	0.0 (0)	0.0 (1)	0.0 (3)	0.0 (91)	0.0 (86)	0.0 (76)

Virus-exposed mosquitoes were incubated for 6, 9 or 12 days. Values in parentheses represent the sample size.

Dissemination rates for DENV-4 virus at 6 and 9 dpe, respectively were 2-fold lower and 3-fold higher among mosquitoes exposed to DENV-4 alone compared to those exposed to both DENV-4 and SINV (Table 3). In contrast, there were no significant differences in dissemination rates between mosquitoes exposed to DENV alone or DENV and SINV at 12 dpe (Table 3). Among mosquitoes exposed to DENV-4 alone, DENV-4 dissemination rates were 4-fold higher at 9 and 12 dpe compared to 6 dpe (Table 3). DENV-4 dissemination rates among mosquitoes exposed to both DENV-4 and SINV were 2- and 3-folds higher at 12 dpe than at 6 and 9 dpe, respectively (Table 3).

There were no significant differences in population dissemination rates between singly and dually infected mosquitoes at 6 dpe (χ^2^ = 0.1, df = 1, *P* = 0.73, Table 3). However, DENV-4 population dissemination rates were significantly higher among singly infected mosquitoes relative to dually infected mosquitoes at 9 and 12 dpe (9 dpe: χ^2^ = 7.5, df = 1, *P* = 0.01, 12 dpe: χ^2^ = 5.3, df = 1, *P* = 0.02, Table 3). Population dissemination rates among singly infected mosquitoes were significantly higher at 9 and 12 dpe compared to 6 dpe (χ^2^ = 8.0, df = 2, *P* = 0.02, Table 3). In contrast, days post exposure had no significant effect on population dissemination rates among dually infected mosquitoes (χ^2^ = 0.5, df = 2, *P* = 0.79, Table 3).

### SINV infection and dissemination rates in *Aedes albopictus*

SINV infection rates were very low ranging from 0-8% among SINV only treatments and 0-4% among coinfection treatments (Table 2). None of the infected mosquitoes had a disseminated infection (Table 3).

## Discussion

The mosquito-borne viruses in *Flaviviridae* and *Togaviridae* families overlap in their geographic distribution and often utilize the same vertebrate hosts and vectors to complete their life cycles. These characteristics increase the likelihood for arboviral coinfections and superinfections in both the vector and vertebrate hosts. For example DENV and CHIKV are principally transmitted by *Ae. aegypti* and *Ae. albopictus* and the range expansion of these vectors and arboviruses has resulted in their geographic convergence and detection of higher number of arboviral coinfections in humans [24,25]. However, the epidemiological implications for vector exposure to coinfections and superinfections with DENV and medically significant members of *Togaviridae* are poorly understood. This study investigated how SINV interacts with DENV-4 in *Ae. albopictus* (C6/36) cell lines and in *Ae. albopictus* mosquitoes. SINV belongs to the same family as the more virulent CHIKV and is readily transmitted by *Aedes* mosquitoes under laboratory conditions. These conditions make it an excellent model for investigating the outcome of *Alphavirus*-*Flavivirus* interactions both *in vitro* and *in vivo*. We found that: 1) SINV inhibited replication of DENV-4 in *Ae. albopictus* cell lines both under coinfection and superinfection conditions, with greater inhibition occurring when the two arboviruses were inoculated simultaneously than sequentially; 2) DENV-4 enhanced replication of SINV when the two viruses were inoculated simultaneously at unequal MOI (1:15 or 15:1) and suppressed it when the two viruses were inoculated simultaneously at equal MOI (1:1); and 3) *Ae. albopictus* mosquitoes were less susceptible to SINV but females that were exposed to DENV-4 alone had significantly higher DENV-4 infection and population dissemination rates compared to those that were simultaneously exposed to DENV-4 and SINV. If these findings apply to medically important *Alphaviruses* that are transmitted by *Ae. albopictus* such as CHIKV, they may imply that prior or simultaneous exposure of this vector species to CHIKV may render it a less effective vector of DENV-4 and perhaps other DENV serotypes.

Interference among arboviruses in invertebrate cell cultures is a well-documented phenomenon. *Ae. albopictus* cell lines persistently infected with SINV were shown to be more resistant to superinfection with both homologous and heterologous *Alphaviruses* but remained susceptible to infection with snowshoe virus, a *Bunyaviru*s [9] and YFV [8]. Similarly, *Ae. albopictus* cells persistently infected with *Bunyamwera* virus were more resistant to superinfection by homologous but not heterologous bunyaviruses [26]. Interference within and among dengue virus serotypes has also been reported [10,11,16]. However, contrary to previous findings where interference was shown to occur only if the interfering virus had an advantage over the challenge virus either in time or MOI [7], coinfection of DENV-4 with SINV had more detrimental effects on DENV-4 replication compared to DENV-4-SINV superinfection treatments. Further, DENV-4 had antagonistic effects on SINV replication when the two viruses were inoculated simultaneously at equal MOI and synergistic effects on SINV replication when SINV was inoculated ahead of DENV at equal MOI or simultaneously with DENV at unequal MOI. Collectively, these findings suggest that interference is a general phenomenon among mosquito-borne viruses but the conditions under which maximum interference occurs may vary among arbovirus systems.

We did not examine the mechanisms responsible for observed viral interferences in cell culture but several mechanisms have been proposed. These include competition for host cell replication sites or substrate necessary for viral replication [7], virus-directed intracellular mechanisms [16], production of defective interfering viral genomes or trans-acting protease by the first infecting virus [8], and exclusion of DENV-4 in cells exposed to both arboviruses such that only a limited number of cells are able to support its replication [9,16]. RNA interference (RNAi) is also believed to be a potential mechanism responsible for viral interference in mosquitoes but C6/36 cells lack a functional antiviral RNAi response [27]. Our experimental design could not allow us to determine which mechanisms were responsible for interference of SINV with DENV-4 replication or interference of DENV-4 with SINV replication in one of the treatments. However, all cells that were infected with SINV either alone or together with DENV-4 had visible cytopathic effects suggesting that competition for replication sites and intracellular host factors may have played a major role in shaping the outcome of interaction between the two arboviruses.

Inhibition of one virus by another in mosquitoes has also been reported previously. *Ae. aegypti* mosquitoes infected with DENV were less susceptible and less capable of transmitting YFV [28]. In vector competence studies involving West Nile Virus (WNV), St. Louis encephalitis, and their natural vector *Cx. quinquefasciatus*, each virus had lower infection and dissemination rates when it was introduced as a superinfection and none of coinfected mosquitoes had a disseminated infection [13]. Rohani et al. [29] demonstrated the failure of CHIKV and DENV to simultaneously replicate in *Ae. agypti*. In contrast, a *Cx. quinquefasciatus* strain from Honduras but not Florida became more competent for WNV when simultaneously inoculated with WNV and *Culex* flavivirus (CxFV) [14]. Moreover, there was no interference between EEEV and Western equine encephalitis in *Culex tarsalis* [30], and *Ae. albopictus* coinfected with DENV-1 and CHIKV was capable of transmitting both viruses in a single bite [31]. In the current study, *Ae. albopictus* females coinfected with SINV and DENV-4, were less susceptible to DENV-4 relative to those infected with DENV-4 alone. Thus it appears that the outcome of virus-virus interactions in mosquitoes may be system specific. Previous studies suggest that interference is more likely to occur between closely related viruses and when the time between mosquito exposure to interfering virus and the secondary virus increases [32,33]. However, our *in vivo* studies corroborated our *in vitro* findings that interference can also occur between distantly related arboviruses and when the two viruses are introduced simultaneously.

When the mosquito ingests a viremic blood meal, the virus enters and replicates in the midgut epithelial cells, and then disseminates to secondary tissues such as fat body, hemocytes, reproductive tissue, legs, nerve tissue, and finally the salivary glands [34]. However, the virus can encounter a midgut infection barrier which restricts its ability to invade the epithelial cells for replication [35] or midgut escape barrier in which the virus is unable to efficiently disseminate from the midgut following efficient replication in the epithelial cells [36,37]. We found that mosquitoes exposed to both SINV and DENV-4 had significantly lower DENV-4 infection rates relative to those exposed to DENV-4 alone suggesting that exposure to SINV enhanced the midgut barrier to infection with DENV-4. Potential mechanisms underlying this form of interference in mosquitoes may include alteration of viral receptors on target cells [38], and RNAi, a proven innate immune pathway by which mosquitoes defend themselves against viruses [39-41].

Unlike in C6/36 cells where both viruses were able to replicate, *Ae. albopictus* populations were refractory to SINV. However, even with the low infectivity, the virus was still able to reduce the susceptibility of this vector species to DENV-4. We have previously used this SINV strain at titers lower than used in the current study to achieve midgut infection rates of between 32 and 43%, and population dissemination rates of between 9 and 20% in the same population of *Ae. albopictus* [21]. We have even obtained a much higher SINV infection and population dissemination rates in *Ae. aegypti* [21,22,42]. Surprisingly, results of this study and other unpublished datasets suggest that our Florida strains of the two vector species have become less susceptible to our stock SINV. Arboviruses may often adapt to features of cell culture that are irrelevant *in vivo* [43] and it is likely that our SINV strain may carry a fixed mutation that has significantly reduced its ability to infect our mosquito colonies. Myles and colleagues demonstrated that a mutation containing a deletion in the E2 glycoprotein reduced infectivity of this virus strain in *Aegypti* while retaining its ability to replicate in cell culture [44]. Alternatively, our mosquito rearing conditions may have selected for mosquito strains that are refractory to this SINV strain. Our future studies will address these hypotheses. If the low infectivity in mosquitoes is driven by deletions in E2 glycoprotein, this may provide the impetus to investigate the potential to utilize this virus strain (SINV MRE16) as a “biocontrol” agent for DENV-4 and other DENV serotypes.

## Conclusion

Taken together, our findings demonstrate that *Ae. albopictus* populations infected with certain *Alphaviruses* (e.g. SINV) may be refractory to dengue virus and could therefore provide an indirect protection against this medically important *Flavivirus*. Competitive displacement among arboviruses has been documented before, mostly among strains of a virus species [45]. For example, the Southeast Asian strain of DENV-2 has displaced the American strain because it is more infectious to the primary vector, *Ae. aegypti* and replicates to a higher titer in humans [46,47]. Similarly, the WNV 02 strain appears to have displaced the NY99 strain in North America [48]. It is still unclear whether our results may be a good reflection of the interactions that may take place between CHIKV and DENV both of which are transmitted by *Ae. aegypti* and *Ae. albopictus* and have previously caused simultaneous outbreaks [49]. Future studies assessing the interaction between CHIKV and DENV in mosquitoes are necessary especially in the light of contradicting findings regarding their potential interactions in the two vector species [29,31]. In addition, studies assessing how arboviral coinfections and superinfections affect other components of vectorial capacity such as vector biting rates and longevity are critical since these components respond differently and sometimes in opposite directions to similar kinds of biotic and abiotic factors [50].

## Abbreviations

DENV: Dengue virus
SINV: Sindbis virus
CHIKV: Chikungunya virus
YFV: Yellow fever virus
LACV: La Crosse virus
EEEV: Eastern equine encephalitis virus
